# Genome Editing by CRISPR-Cas: A Game Change in the Genetic Manipulation of *Chlamydomonas*

**DOI:** 10.3390/life10110295

**Published:** 2020-11-20

**Authors:** Manel Ghribi, Serge Basile Nouemssi, Fatma Meddeb-Mouelhi, Isabel Desgagné-Penix

**Affiliations:** 1 Department of Chemistry, Biochemistry and Physics, Université du Québec à Trois-Rivières, 3351, Boulevard des Forges, C.P. 500, Trois-Rivières, QC G9A 5H7, Canada; Manel.Ghribi@uqtr.ca (M.G.); Serge.Nouemssi@uqtr.ca (S.B.N.); Fatma.Meddeb@uqtr.ca (F.M.-M.); 2 Groupe de Recherche en Biologie Végétale, Université du Québec à Trois-Rivières, 3351, Boulevard des Forges, C.P. 500, Trois-Rivières, QC G9A 5H7, Canada

**Keywords:** CRISPR-Cas, *Chlamydomonas*, genetic engineering, green microalgae, genome editing

## Abstract

Microalgae are promising photosynthetic unicellular eukaryotes among the most abundant on the planet and are considered as alternative sustainable resources for various industrial applications. *Chlamydomonas* is an emerging model for microalgae to be manipulated by multiple biotechnological tools in order to produce high-value bioproducts such as biofuels, bioactive peptides, pigments, nutraceuticals, and medicines. Specifically, *Chlamydomonas reinhardtii* has become a subject of different genetic-editing techniques adapted to modulate the production of microalgal metabolites. The main nuclear genome-editing tools available today include zinc finger nucleases (ZFNs), transcriptional activator-like effector nucleases (TALENs), and more recently discovered the clustered regularly interspaced short palindromic repeats (CRISPR)-CRISPR associated protein (Cas) nuclease system. The latter, shown to have an interesting editing capacity, has become an essential tool for genome editing. In this review, we highlight the available literature on the methods and the applications of CRISPR-Cas for *C. reinhardtii* genetic engineering, including recent transformation methods, most used bioinformatic tools, best strategies for the expression of Cas protein and sgRNA, the CRISPR-Cas mediated gene knock-in/knock-out strategies, and finally the literature related to CRISPR expression and modification approaches.

## 1. Introduction

Microalgae or microphytes are well-known microscopic photosynthetic algae, typically found in the freshwater and marine systems. In addition to their ability to fix atmospheric CO_2_ through various metabolic pathways, these fast growing green biofactories can produce high-quality biomass enriched with various products for industrial uses (fuel, food, cosmetics, pharmaceutics). Compared to land plant-based photoproduction, microalgae do not compete with agriculture and can be grown at high yields in large-scale setting [1]. Among microalgae species, the green eukaryotic unicellular biflagellate microalga, *Chlamydomonas reinhardtii*, is a well-established model organism for basic and applied research [2]. Supporting research into various aspects of *C. reinhardtii* is available at the *Chlamydomonas* Resource Centre (www.chlamycollection.org). Numerous resources are available including well-defined protocols for growth, sexual propagation, and mutagenesis, as well as numerous strains, plasmids and published biochemical, analytical and reporter assays. *C. reinhardtii*‘s life cycle has been characterized and sequences for the nuclear, chloroplastic, and mitochondrial genomes have been completed. Specifically, the larger nuclear genome is of 110 Mb containing 17 chromosomes with approximately 17,000 genes annotated whereas the chloroplastic genome (203.8 Kb comprising 99 genes) and the mitochondrial genome (15.8 Kb with 13 genes) are much smaller [3].

The presence of the three different genomes along with numerous molecular and genomic resources support *C. reinhardtii* as a promising biotechnological platform for basic and industrial applications. Several advanced techniques have been used to improve the microalgae biomass, bio-products or biofuels production but recently several researchers favor genome editing to enhance the capabilities of microalgae. The challenge in the genetic engineering of microalgae is the need for efficient methods to specifically knock-down or knock-out unwanted genes in the nucleus.

Thus, the development and expansion of a biotechnology toolbox (set of various techniques for inserting, adding, removing or editing genes) for *C. reinhardtii* represent a powerful and major advance for genome engineering and other biotechnology applications for *Chlamydomonas* and potentially for other green microphytes [4]. The knock-in involves the one-for-one substitution of DNA sequence information in a specific genetic locus or the insertion of DNA sequence not found previously within the locus. This will induce the expression of the new substituted gene. In contrast with knock-out, this genetic engineering technique aims to either delete totally or partially a DNA sequence existing in a specific locus which will disrupt its expression. When the gene is modified and its expression is reduced, this is called a knock-down. 

In addition, the genetic engineering of *C. reinhardtii* remains a vital strategy to prevail constraints related to mass production and silencing endogenous harming genes, which requires the editing or insertion of exogenous genes. Indeed, having fully sequenced genomes bestow a complete genetic background and provide strong impetus for the engineering of this microalga [5]. Although *C. reinhardtii* is widely used in basic and applied research, its use has been limited by difficulties in modifying or inactivating genes (silencing) by directed gene targeting. Thus, to further develop microalgae as robust systems for the production of valuable metabolites, the elaboration of an effective toolbox is a necessity.

Understanding *C. reinhardtii* nuclear genetics, has also motivated researchers to work toward the creation and the optimization of transgenic *C. reinhardtii* strains for better expression of industrial products [1]. Genome editing has become possible with the development of molecular technologies, such as RNA/DNA gene repair, zinc finger nucleases (ZFNs), transcriptional activator-like effector nucleases (TALENs), and clustered regularly interspaced short palindromic repeats-CRISPR-associated protein (CRISPR-Cas) [6]. Although each method has its advantages and disadvantages, CRISPR-Cas9 has become the system of choice because of its versatility, adaptability, and ease of use. Indeed, homologous recombination (HR) between endogenous DNA and homologous DNA templates in *C. reinhardtii* is rare because the double-stranded breaks (DSBs) if they occur are in most cases repaired via error-prone non-homologous end joining (NHEJ) rather than high-fidelity homology-directed repair (HDR), with no controlled number of copies and no knowledge of the insertion location, especially in genomic DNA [7]. Thus, impeding targeted gene modifications. 

CRISPR-Cas9 provides sequence-specific recognition tool as well as a simple technique to produce sequence-specific genomic breaks. In the past decade, CRISPR-Cas systems have transitioned from an adaptive immune defense system in bacteria and archaea to revolutionary genome-editing tools used in various organisms, from bacteria to plants and animals including humans [8]. The resulting CRISPR technologies have driven innovations for treating genetic modification and device to another dimension by modifying diverse eukaryotic genomes [9]. Also, several microalgae species have been modified with CRISPR-Cas genome editing such as *Nannochloropsis oceanica* [10], *Phaeodactylum tricornutum* [11], *Thalassiosira pseudonana* [12], and *Chlorella vulgaris* [13]. The current review solely focuses on the green algae *C. reinhardtii*. 

Despite the simplicity of microalgae cells, few of the CRISPR-Cas-mediated transformation and genetic editing have been successful ([14,15,16]). Through all the attempts and trials to edit target genes in microalgae, many pitfalls have been encountered harming and slowing down the research in this field. For example, one of the pitfalls is the off-target effect and repair mechanisms that make microalgae genetically unstable that will be discussed later in this review. Moreover, DNA mismatches are responsible for off-target cleavages that are unable to “lock” the protein domain of the Cas protein, thereby leading to the unselective cleavage of DNA sequences.

Regarding *C. reinhardtii*, with a fully sequenced and annotated genome (version V5.5 [17]), different CRISPR-Cas-systems-mediated transformation have been reported. The first attempt to modify target genes in the genome of *C. reinhardtii* was made in 2014 [18]. Unfortunately, the implementation of CRISPR-Cas9 in *C. reinhardtii* was not as straightforward or as efficient as in other model organisms. Previous studies showed highly inefficient gene editing in *C. reinhardtii* using conventional Cas9 and single guide RNA (sgRNA) genes which yielded only one colony with a modified target locus per 1.5 × 10^9^ initial cells used for transformation [18]. From there, several research teams have developed different strategies and methods to transform *C. reinhardtii* cells either by experimenting with CRISPR systems [19], transformation techniques [20], and even by modifying CRISPR-associated protein used for the purpose [21].

In 2016, innovative transformation methods were applied to *C. reinhardtii* where two main research groups focused on the use of DNA free CRISPR systems for algae transformation [15,22]. From there different methods and proof of concept have been implemented for the transformation of *C. reinhardtii*. Visual phenotypic observation (e.g., color) of algae colonies has been the leading strategy in those studies [18]. For example, genes involved in pigment production conferring algae green color were examined as a clear proof of concept for the success or the failure of the algae CRISPR transformation [14,15]. After targeting these “pigment” genes, the selection of colonies was done by visual observations and the color obtained may vary from dark green to pale green, yellowish and white colonies when phytoene synthase 1 (*PSY1)* gene was knocked-down [14] or the chloroplastic *FTSY* (*CpFTSY)* gene was disrupted [15]. This rapid and convenient visual screening method was successful to validate the knock-out (total or partial deletion of a DNA sequence existing in a specific locus which will disrupt its expression) or knock-down (the gene sequence is modified and its expression is reduced) of targeted genes. Recently, a study using CRISPR-Cas9 to enhance dietary carotenoids in *C. reinhardtii* cells reported significant fold increase [23]. However, only a few applications have been reported for the CRISPR-Cas system transformation in microalgae to produce molecules of interest. In addition, no CRISPR-transformed *C. reinhardtii* strains have been described for scale-up applications.

Altogether, these studies showed that different factors and parameters need to be considered for successful CRISPR modification in *C. reinhardtii*. In this review, we highlight the available literature on the methods and the applications for CRISPR-Cas for microalgal genetic engineering. The description will include the latest transformation methods, a summary of the most used bioinformatics tools, strategies for the expression of Cas proteins and sgRNA, the CRISPR-Cas9-mediated gene knock-in/knock-out methods, off-target and on-target effects, and CRISPR modification strategies. We aim to provide a toolbox of information for CRISPR-Cas modifications of *C. reinhardtii*.

## 2. Summary of the Pipeline for Using the CRISPR-Cas System for Genome Editing in Green Microalgae *C. reinhardtii*

**Figure 1:**
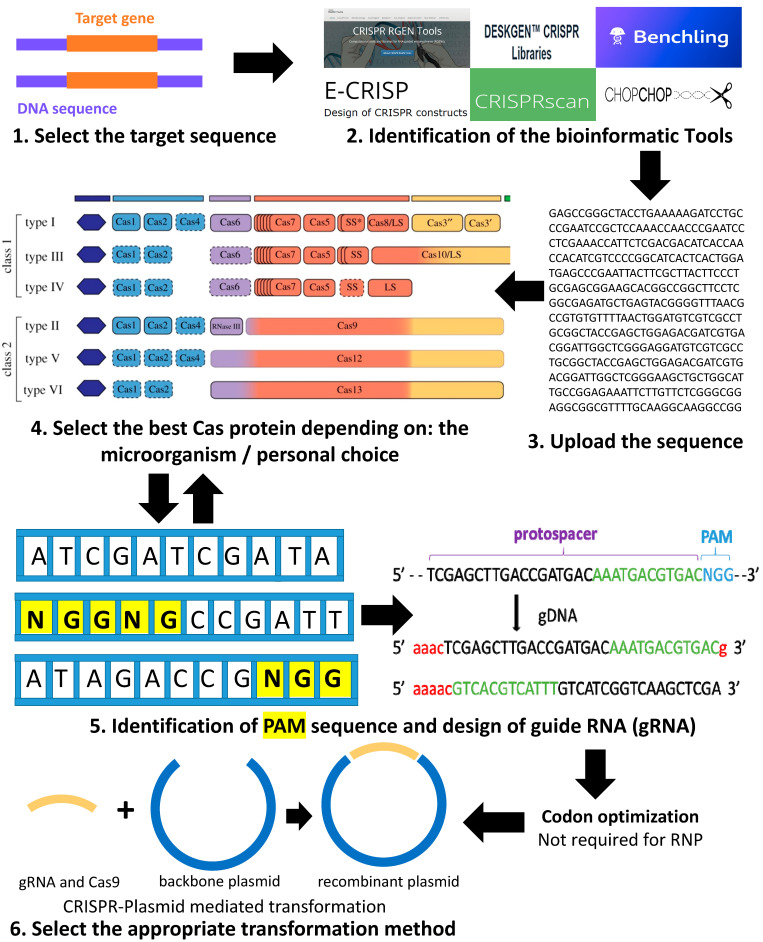
Important steps for the design of sgRNA (adapted from [24]). **1**: Select the target gene sequence. **2**: Identify the bioinformatic tool suitable for the organism (here, *C.* *reinhardtii*). **3**: Upload the sequence of the gene of interest and select the organism (here in *C. reinhardtii)* in the database of the software. **4**: Select the best Cas protein to be used or in some software the PAM sequence. **5**: The software will automatically choose the adequate Cas protein identified by the user and its locations in the genome will be indicated by different colors or arrows. Once the PAM (highlighted in yellow) is selected, the user can choose the option design guide RNA and save the sequence. Then, Cas protein and gRNA can be codon optimized for better protein expression. **6**: Select the proper CRISPR system for the transformation (either plasmid-mediated CRISPR or RNP-mediated CRISPR).

CRISPR-Cas9 is a simple two-component system that allows researchers to accurately edit any sequence in the genome of an organism. This is achieved by the guide RNA, which recognizes the target sequence, and the CRISPR-associated endonuclease (Cas) that cuts the targeted sequence. A brief introduction on the order of events to use in the design of a CRISPR knock-out or knock-down system in *C. reinhardtii* is presented in Figure 1. Details for each step are described in the following sections. The first step starts with the selection of the target gene, preferably a stable gene without multiple copies in the desired genome. Next comes the choice of the software to use and adapted to *C. reinhardtii*. The third step includes the design of the guide RNA by uploading the target gene sequence into the software and it also involves selecting the adequate Cas protein for the work (selected from the list usually generated by the software and depending on personal choice). The software will help to choose the appropriate PAM (protospacer adjacent motif) and will generate different single guide RNA (sgRNA) with highlighted PAM (Figure 1). The PAM is a short DNA sequence (usually 2–6 nucleotides in length) that follows the DNA region targeted for cleavage by the CRISPR system. The PAM is required for the Cas nuclease to cut and is generally found 3–4 nucleotides downstream from the cut site. Then, we can select the most suitable sgRNA for the desired project (with the minimum of off-target loci if possible).

Once the appropriate sgRNA, Cas protein, and targeted gene are selected, comes the selection of the transformation method. Select the proper CRISPR system for the transformation of either plasmid-mediated CRISPR or RiboNucleic Protein-CRISPR (RNP-CRISPR). If the RNP-CRISPR is selected, the optimization of Cas9 and synthesis of guide RNA can proceed with or without codon optimization. However, if the plasmid-mediated transformation is selected, the user should choose a backbone plasmid in which he has to clone or synthesize the sequence of Cas and gRNA. Then, optimize the codons and synthesize the sequences of Cas protein and sgRNA.

### Codon Optimization

The lack of compatible parts between microorganisms is one of the most important reasons for the expansion of synthetic biology tools, especially codon optimization. Even though foreign genes are easily integrated into *C. reinhardtii*, the transgene expression is often weak [25]. The efficiency of the gene expression strongly depends on the nucleotide identity (rich or poor on GC content, especially at the wobble position (3rd letter of codon)) and depends on the usage of the codon by the microalgae [26]. The codon sequences (CDSs) of *C. reinhardtii* have an average of 68% GC content [27]. By consequence, a slight change in the GC content in *C. reinhardtii* codons drastically affects the gene expression at the chromatin level.

It is worth mentioning that previous data of codon usage comes from the web site Kazusa [28] and is calculated from 846 CDSs. However, nowadays, different software and companies are doing rapid codons optimization for *C. reinhardtii* sequences on the basis of the codon adaptation index (CAI). The CAI of each gene is computed as the geometric mean of the relative adaptiveness of all the codons in the coding sequence [26]. It is also worth highlighting that the addition of intron-containing sequence can enhance the expression in *Chlamydomonas* [29]. It might be an avenue in the improvement of its toolbox.

Beside the genetic cassettes (sgRNA) to be inserted, some of the Cas9 protein sequences used in previous cited articles were also codon optimized. Thus, different *C. reinhardtii* cell type has been modified genetically in regard of this tool. However, some issues regarding the efficiency of transformation have been encountered. The construction of a proficient and mature CRISPR-Cas expression system is now essential for efficient targeting.

## 3. CRISPR-Cas System in *C. reinhardtii*

### 3.1. Cas/Cas9 Protein and Guide RNA

#### 3.1.1. Definition

The CRISPR-associated protein, or Cas protein, is an endonuclease protein guided by its own localization system—GPS, i.e., the single guide RNA (sgRNA) to the active zone of the targeted site for the break of double-stranded DNA of interest [30]. Cas proteins belong to the immune system of bacteria, to protect them from foreign nucleic acids, such as viruses or plasmids, where it was first discovered. In addition to its bacterial function in the immunity system, Cas9 protein is now used as a genetic engineering tool to induce double-stranded breaks (DSBs) at target DNA site. These ruptures can lead to gene inactivation (knock-out) or the introduction of heterologous DNA through two mechanisms of repair: the junction of NHEJ and HR in many model laboratory organisms (Knock-in) [31]. The knock-in could involve the one-for-one substitution of DNA sequence information in a specific genetic locus or the insertion of DNA sequence not found previously within the locus. This will induce the expression of the new substituted gene.

Of the 497 non-redundant Cas proteins identified to date, only Cas9 and its variant protein Cpf1 (also known as Cas12) were used for the genetic editing of *C. reinhardtii*.

**Figure 2:**
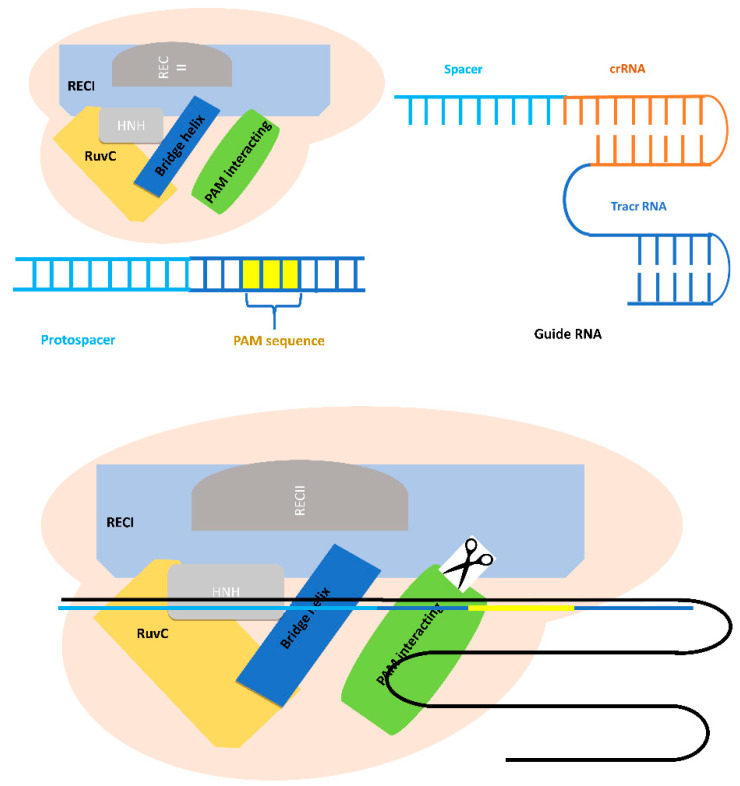
Schematic structure of Cas9 protein of the type II CRISPR-Cas system and the guide RNA and how it binds to the targeted DNA sequence.

As mentioned above, the Cas9 protein is guided by its own sgRNA. As the name implies, a sgRNA is a single RNA molecule that contains both the custom designed short CRISPR RNA (crRNA) sequence fused to the scaffold trans-activating CRISPR RNA (tracrRNA) sequence. sgRNA can be synthetically generated or made in vitro or in vivo from a DNA template (Figure 2). While crRNAs and tracrRNAs exist as two separate RNA molecules in nature, sgRNAs have become the most popular format for CRISPR guide RNAs for researchers, so the sgRNA and gRNA terms are often used with the same meaning in the CRISPR community these days [32]. 

Cas9 protein has six domains: RECI, RECII, Bridge Helix, PAM Interacting, HNH, and RuvC (Figure 2). The HNH and RuvC domains are nuclease domains that cut single-stranded DNA that are complementary and non-complementary to the 20 nucleotides (nt) RNA guide sequence in CRISPR RNA (crRNAs) [33]. The RuvABC protein complex is involved in the Holliday junction (phenomena happening during recombination, when two double-stranded DNA molecules become separated into four strands in order to exchange segments of genetic information) during the homologous recombination in bacteria, for example. The most important domain of RuvABC is the RuvC protein. This latter is required to cleave the DNA [34].

Table 1 and Table 2 present a summary of the most important Cas proteins and their characterization regarding their microorganism source, PAM specific sequences, etc., as well as the recently developed support tools (online open sources) designed to create the sgRNA.

As described in Table 1, the most used Cas proteins in *Chlamydomonas* are: SaCas9 (*Staphylococcus aureus* Cas9), StCas9 (*Streptococcus thermophilus* Cas9), SpCas9 (*Streptococcus pyrogenes* Cas9), and Cpf1 (Cas from *Prevoltella* and *Francisella 1*). Furthermore, other Cas proteins like dCas9 (dead Cas9) or nCas (Nikase Cas9) have not been adapted yet in *Chlamydomonas* CRISPR system. However, it could be interesting to test those Cas proteins in the green microalgae *C. reinhardtii* to elucidate their mechanism and efficiency.

The most suitable open-access software for *C. reinhardtii* from the previous table are Cas-OFFfinder, CHOP-CHOP, E-CRISP, and Benchling. Unfortunately, this is all the database available for *C. reinhardtii* until now. However, it is worth mentioning that these bioinformatic tools are constantly updating to increase the genomes’ variety in their database.

Throughout various studies in the CRISPR system, researchers have noticed that many factors other than Cas9 influence CRISPR gene-editing performance. Below, some of these factors that should be well considered when designing sgRNA are summarized.

#### 3.1.2. Regarding the Off Target and On Target Efficiency

Initially, CRISPR-Cas9 was thought to be able to target any 20 base-pair sequence that was flanked by a PAM. Different Cas and related enzymes target different PAMs, and there is ongoing research into designing enzymes with specific PAM recognition ability ([52,53]). However, the most used SpCas9, and the focus of this review, targets an NGG motif. As such, early tools for target site selection were simple pattern recognition programs that identified instances of this motif [54]. The off-targeting genome editing is a non-specific and non-voluntary genetic modification that occurs in the genome after the activation of the Cas proteins. These endonucleases arouse the creation of a double-stranded DNA break (DSB). The cleavage of the DNA in question summons the cell’s DNA repair mechanism NHEJ and HR (these two concepts are discussed later in this review) and leads to site-specific modifications [55]. In case that the complex (sgRNA and Cas9) does not bind to the predetermined DNA and also goes to cleave homologous sequences generating mismatch tolerance in the cell, the CRISPR-Cas system could be considered as cleaving off-target which may generate non-specific genetic modifications [56]. In *C. reinhardtii*, most of the off-target genetic modification consists of unintended point mutations. However, these targeted genetic mutations may vary from insertion, inversion, and even translocation. At the experimental level, the off-targeted effect may lead to non-reproducible results. Many of the bioinformatics tools cited in Table 2 are working to improve the off targeted detection percentage. It is crucial for any organism or when designing a CRISPR system for *C. reinhardtii* to make sure to choose the less off-target effect. 

#### 3.1.3. The Case of the Cpf1 Protein

Zetsche et al. [38] found that Cpf1 (Cas protein from *Prevotella* and *Francisella* 1) protein might be more convenient in gene editing than the Cas9 system because it only needs mature CRISPR RNAs (crRNAs) for targeting. In contrast, the CRISPR-Cas9 system requires both tracer CRISPR RNA (tracrRNA) and CRISPR RNA (crRNA). T-enriched PAM and staggered DSB at the end of the spacer are notable features in Cpf1, bringing new options in experimentation [38]. So, in this case, it is not recommended to design the whole guide RNA but only the crRNA when using Cpf1. Also, as mentioned in Yang et al., 2019 [57], Cpf1 has better efficiency in the GC-rich genome than Cas9 protein. However, this might apply to *Chlamydomonas* and probably could explain why its use in *C. reinhardtii* genome has higher efficiency than Cas9. 

#### 3.1.4. Dead Cas9

Dead Cas9 protein is a completely inactive nuclease that binds to DNA, but it is not able to induce its cut. They are Cas proteins that are devoid of nucleolytic activity, and they can be used to deliver functional cargo to programmed sites in the genome. In CRISPR activation, the dCas9 binds to gRNA and still targets the DNA and can activate the transcription. On the other hand, CRISPR interference still enables the complex (sgRNA and dCas9) to bind to the targeted DNA, but it blocks its transcription [58]. As far as we know, dCas9 was used for *C. reinhardtii* as toxicity’s control of the Cas9 only in the study of Jiang et al., 2014 [18].

#### 3.1.5. Nickase Cas9

Ran et al., 2013 [59] devised a “double-nicking” approach by using catalytic mutant Cas9D10A nickase to minimize off-target effects. The Cas9 D10A nickase mutation inactivates the RuvC domain, so this nickase cleaves only the target strand, effectively reducing the off-target effects. In this gene-editing approach, two modified effectors will bind to two opposite strands and surround 10 to 31 nt target sequence and then make a DSB at the DNA in the target site. 

#### 3.1.6. The Size of sgRNA

Truncated sgRNAs with 17 to 19-nt spacer are more sensitive to mismatches than others with 20-nt length, which can effectively reduce off-target mutations. Besides, the recent study [60] has shown that shorter spacers such as 17 to 18 nt have higher specificity but perform less on-target effective than 19 or 20-nt spacers. Thus, the choice here is tough to design the best sgRNA guide sequence without creating mismatches. As it is a relatively new tool in *C. reinhardtii*, testing different gRNA sizes could be, therefore, a solution.

### 3.2. Current CRISPR-Cas Systems in C. reinhardtii 

**Figure 3:**
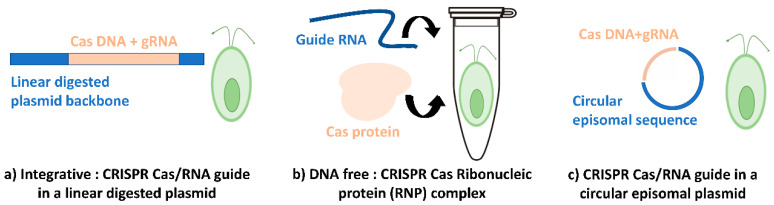
The different systems reported regarding CRISPR-Cas integration. (**a**) Linearized plasmid during non-homologous genetic integration. (**b**) DNA free (RNP-CRISPR) with no integration in the genome. (**c**) Episomal plasmid remaining circular while transformation in *C. reinhardtii* cells.

CRISPR-Cas is now one of the most used genome-editing tools for eukaryotic cells. There are few different CRISPR systems valued for *C. reinhardtii*. As far as we know, two CRISPR systems have been adopted for *C. reinhardtii*: the (1) Integrative CRISPR-Cas system and (2) Ribonucleic Protein CRISPR (RNP-CRISPR) or DNA free CRISPR (Figure 3a,b).

Another promising system is c) the episomal CRISPR vector that only has been tested in the green microalgae *Nannochloropsis oceanica* [10]. Muñoz et al., 2019 showed stable transformation of *Acutodesmus obliquus* and *Neochloris oleoabundans* using *E. coli* conjugation [61]. The latter belongs to the green algae family, so we assume it could be adaptable and useful for *C. reinhardtii* (Figure 3c).

#### 3.2.1. Integrative CRISPR System

The main bottleneck for the genetic modification of microalgae is the limited genetic toolbox that is currently available. Therefore, the implementation of CRISPR-Cas9 in *C. reinhardtii* has not been as straightforward and as efficient as in other model organisms. In 2014, the first attempt at implementing the CRISPR system in *C. reinhardtii* yielded only one colony with a modified target locus per 1.5 × 10^9^ initial cells used for transformation. This extremely low efficiency was attributed at that time to Cas9 being potentially toxic to the cells [20]. This study, considered at first discouraging, has been followed within two years by a successful transformation of the CRISPR-Cas system in *C. reinhardtii* cells by electroporation transformation method. This study targeted the genes of the light-harvesting complex and yielded in algae with pale green color as described by Baek et al., 2016 [15].

The use of circular recombinant plasmid for CRISPR transformation has been a standard method at the dawn of related research. This plasmid contains the *Cas9* gene encoding for its protein and sgRNA that will be hybridizing with the target gene with the backbone of whatever vector works best for *C. reinhardtii* (Figure 3a). After that, the plasmid will be digested and linearized with a restriction enzyme(s) and integrated into the microalgae’s genome via the transformation method (usually electroporation). Cas9 protein—guided by its sgRNA—will induce then the DSB (double-strand break) and thus ensure the targeted gene’s disruption.

#### 3.2.2. RNP DNA Free CRISPR System

One alternative approach to avoid many of these complications is the use of ribonucleoprotein (RNP)-mediated CRISPR genome or the also called DNA-free CRISPR. This direct delivery of RNPs alleviates difficulties with protein expression in cells where common eukaryotic promoters are not expressed. Indeed, this method does not require the delivery of foreign DNA. So, the Cas9-gRNA RNP can be degraded over time and using RNPs may limit the potential for off-target effects (Figure 3b). In 2016, this delivery method was used to obtain mutants for phenotype screening in *C. reinhardtii* ([15,22]). A year later, Greiner’s team [14], was able to modify the Cas9 gRNA RNP technology to allow the use of their *C. reinhardtii mut-aphVIII* gene repair assay. They transformed their previous mutants *mut-aphVIII* strain with EMX1 SpCas/gRNA RNPs (contains target sites for the well-characterized human *EMX1 homeobox protein* gene) using homology direct repair HDR-repair donor (*pHDR-APHVIII∆120*) and they obtained ~300 colonies with EMX1 SpCas9/gRNA RNPs per 4 × 10^6^ electroporated cells. Greiner and his team also demonstrated that the increase in the temperature from 22 to 33 °C for recovery after transformation did not alter the efficiency, unlike in the plasmid-based system. One major pitfall of this CRISPR system is that optimizing the quantity of sgRNA and Cas protein when mixed should be optimized, and the ratio should be determined. Their incubation time should be determined (varying from 10, 15, and 20 min). Nevertheless, this method stays by far the most time saving and efficient, and it also generates non-transgenic strains.

#### 3.2.3. Episomal CRISPR System

Karas et al., 2015 [62] and Diner et al., 2016 [63] showed that episomal plasmids containing a yeast-derived centromeric sequence *CEN6-ARSH4-HIS3* could be transferred by conjugation from *E. coli* strains (e.g., Epi300) to the diatoms *Thalassiosira pseudonana* and *Phaeodactylum tricornutum*. When centromeric sequences *CEN9-ARSH4-HIS3* are not present in the plasmid of interest, the previous studies showed a random integration of the plasmid in the nuclear genome of those microalgae, precisely as an integrative transformation. In 2019, Munoz and his team [61] reported the first efficient and stable transformation of the green microalga *Acutodesmus obliquus* and *Neochloris oleoabundans*. In Munoz’s study, the conjugation with *E. coli* containing the vector of interest was the used method for transformation. Although successful transformations were achieved, they were not able to recover the plasmid from the positive transformants, which raises many questions regarding the use of episomal vector into the green microalgae (Figure 3c). However, the oleaginous microalga, *Nannochloropsis oceanica*, has been successfully transformed by one episomal vector containing CRISPR-Cas9 system that enables the generation of marker-free mutant lines. This system efficiently generates targeted mutations and allows the loss of episomal DNA after removing the selection pressure, resulting in marker-free non-transgenic engineered lines. In the study reported by Poliner et al., 2019 [16], the *nitrate reductase* gene (*NR*) has been disrupted and subsequently, the CRISPR episome has been successfully removed to generate non-transgenic marker-free nitrate reductase knock-out lines (NR-KO).

The use of episomal circular non-transgenic vector carrying the Cas9 protein was never experimented as an approach to generate a knock-out in *the C. reinhardtii* genome. Nevertheless, the development of a plasmid encompassing episomal sequence as well as another plasmid-containing Cas protein and a guide RNA responsible for the DSB of targeted genes in green microalgae [61], are both indicators that we are moving forward with the development of episomal CRISPR-Cas vector in *C. reinhardtii.*

## 4. Repair System in *C. reinhardtii*

### 4.1. NHEJ

NHEJ is the predominant DSB repair pathway throughout the cell cycle and accounts for nearly all DSB repair. It is also known for being an error-prone system as it brings nucleotides randomly to repair the DSB. 

NHEJ is a single pathway with multiple enzymes at its disposal to repair DSBs, resulting in a diversity of repair outcomes. NHEJ system to repair the (DSBs) in DNA is a task accomplished by several vital proteins that work together to ensure the ligation, preparation, and synapsis of the broken DNA ends. The main proteins involved in NHEJ in eukaryotes are Ku protein (heterodimer consisting from Ku70 and Ku80, working as a toolbelt for all the other proteins docking), DNA ligase IV, and XRCC4 (the catalytic subunit of DNA-dependent protein kinase (DNA-PKcs)) [63].

In *C. reinhardtii*’s case, all these proteins have been identified, and the NHEJ repair system is the most active in this microalga.

There are mainly three essential steps that have been identified in the NHEJ process [64]:

(i) DNA end-binding and bridging,
(ii) Terminal-end processing,
(iii) Ligation.

#### 4.1.1. (i) DNA End-Binding and Bridging

The NHEJ is launched by the recognition and ligation of the Ku protein to the broken DNA ends. This protein is known for being a heterodimer of the Ku70 and Ku80. Together they form the DNA-binding component of protein kinase (DNA-PK). The protein Ku forms then a ring that encircles duplex DNA. Indeed, this protein proceeds by structurally supporting and aligning the DNA ends. This leads to the forming of a bridge between the broken DNA ends. Thus, Ku aligns well with the DNA, while still allowing access of other repair protein (polymerases and ligases) to the broken DNA ends to promote end joining [65].

Once Ku is settled, it requires the catalytic subunit of DNA-PK (phosphoinositide 3-kinase (PI3K)) family. However, this mechanism has not been studied in detail in *C. reinhardtii*. In this review it is assumed that *C. reinhardtii* may follow the same repair system steps as in eukaryotic cells in general.

#### 4.1.2. (ii) Terminal End Processing and (iii) Ligation

Ku also recruits a specific DNA ligase (DNA ligase IV for eukaryotes), which sutures the strands by reforming the cleaved phosphodiester bonds. When the ends of the strand break are not definite, the Artemis protein will remove some bases. Its endonuclease activity is supported by the terminal transferase and will make it possible to ligate the strands with DNA ligase [66]. During this so-called “end process”, certain bases can be destroyed and thus lead to mutations more known as “indels” for insertions, deletions, or frameshifts.

### 4.2. HDR

Homology directed repair (HDR) is a mechanism induced into the cells to repair DSB lesions. The HDR form that happens the most in eukaryotic cells is homologous recombination. The HDR mechanism occurs only when there is a homologous sequence of DNA that is present in the genome of the organism of interest. This repair can be performed either by a synthetic template donor plasmid or by the presence of a new single strand of DNA (ssDNA), encompassing homology arms [67].

The use of the ssDNA approach seems to be way easier than the plasmid template. The ssDNA is a synthetic DNA that can integrate into the place of DSB. The ssDNA length should not exceed hundreds of bp when the homology arms (left and right) are about 30 bp each. This leaves around 40 bp for the insert to get into the DSB, which makes this approach not very suitable when the insert’s size is more significant than 40 bp. But it may work well as proof of concept of a possible happening repair in *C. reinhardtii* cell [68]. In fact, Ferenczi et al., 2017 [21] were the first to report the use of CRISPR/Cpf1-mediated DNA-editing efficiencies being boosted 500-fold through the use of single-stranded oligodeoxynucleotides (ssODNs) as repair templates. It remains to be determined whether Cpf1-induced staggered DNA cleavage enhances ssODN-mediated gene editing in a wider range of species and whether the underlying repair pathway(s) responsible is more broadly conserved.

For dsDNA, it is usually a plasmid that encompasses the repair cassette. The repair cassette or insert has no limit in report size to be efficiently compared to other eukaryotic cells and previous related literature [69]. The left and right homology arms should be around 1 Kb each to ensure a better integration with the DSB sequence. It is also interesting to use any backbone plasmid that already worked for *C. reinhardtii* (e.g., pHDR-*aphVII*I), as used by Greiner et al. [14], and try to re-integrate it as a backbone of the donor in the HDR.

## 5. Transformation Methods

A key step of any CRISPR workflow is delivering the gRNA and Cas9 into the target cells’ cytoplasm or nucleus. Different transformation techniques have been developed for micro-algae such as chemical, physical/mechanical, and biological methods [70]. Most of these approaches are based on creating permeability in the green microalgae membrane/cell wall, which enables the CRISPR-DNA to penetrate the cell and facilitates its integration into the target locus to occur.

The introduction of foreign DNA into the green microalgae cells is not a tremendous task. However, the cell’s recovery from the damage caused by the administration of exogenous CRISPR-DNA or the disruption of the gene of interest, remains the most critical part determining a successful transformation.

To deliver enough amounts of exogenous DNA into cells, an optimal transformation method is inevitable. Electroporation [71], glass beads [72], particle bombardment [73], and *Agrobacterium tumefaciens*-mediated transformation [74] are the main transformation methods used in recent years for *C. reinhardtii*. However, for CRISPR, successful integration has only occurred in this microalga via electroporation and glass beads methods. No *Agrobacterium-*mediated system, silicon carbides, or biolistic transformation have been valued for the CRISPR system in *C. reinhardtii* cells.

### 5.1. Electroporation

As cited previously, this technique is the top optimal method for *C. reinhardtii* cells transformation, as it is swift and does not require high DNA quantity, and is also very efficient in the number of generated transformants [75].

In this transformation method, the reported size of the plasmid used is up to 14 Kb. Electroporation can also be used for the free CRISPR-RNP complex and *C. reinhardtii* cells used for transformation can be with and without intact cell walls.

Several factors influence the electroporation’s success, including field strength, pulse length, medium composition, temperature, and the membrane characteristics of the particular cell type. It is reported that the transformation efficiency with this method is around 10^5^ transformants per μg of exogenous DNA [71]. However, when using electroporation for CRISPR transformation, the efficiency of transformation is far less because of the NHEJ repair system in *C.* *reinhardtii*, as mentioned in [14] and/or because of recovery issues. In general, the quantity of needed CRISPR-DNA or DNA for electroporation is usually in the order of hundreds of ng and rarely above 1 µg [15].

Recently, an improved electroporation technique was applied for wild-type cells without cell-wall removal by the square electric pulse generating electroporator, which yields approximately 0.4–3 × 10^3^ transformants per μg of exogenous DNA using 400 ng of DNA per trial [76].

After electroporation, *C. reinhardtii* cells suspension should be transferred into a conical centrifuge tube containing TAP medium with 60 mM sorbitol/ sucrose for overnight recovery at dim light by slowly shaking. After overnight recovery, cells were collected by centrifugation and were plated onto 1.5% (*w/v*) agar TAP media with adequate antibiotics to obtain single colonies for further investigation. It is crucial when using a CRISPR-Cas system to be careful about any disturbance of the cells in terms of light or temperature (low-moderate 50 µE/m/s and 25 °C are recommended), as cells are very fragile where the CRISPR system should integrate and start its action correctly.

### 5.2. Glass-Beads

This technique is a short and relatively cheap method for the non-homologous nuclear transformation of *C. reinhardtii*. Unlike the electroporation, only one previous study reported using the glass beads method for CRISPR transformation in *C. reinhardtii* [77]. This method works well with linearized DNA. It requires glass beads for DNA transformation, usually, acid-washed glass beads. The latter are added to *C. reinhardtii* cells in the presence of PEG (polyethylene glycol 8000) and the foreign CRISPR-DNA. This mixture should be vortexed for a few seconds at a few repeats. 

Once the beads could settle, cells were collected for recovery in liquid TAP media, then spread on selective agar plates and finally incubated from 7 to 10 days or until the appearance of transformants. One inconvenience of this method is the quantity of the required DNA. It should be a minimum range in the order of µg. Indeed, this method is beneficial for plasmid with a size under 10 Kb and is efficient, generating between 10^4^ to 10^6^ cells per 1µg of non-CRISPR-DNA. Always keep in mind that with CRISPR transformation, the efficiency of transformation was low [77].

### 5.3. Biolistic

Biolistic devices are initially made for chloroplast transformation in algae [78]. Stable transformation of the chloroplast was first achieved in 1988, using the newly developed biolistic method of DNA delivery to introduce cloned DNA into the genome of the green algae *C. reinhardtii* [79]. This tool biological-ballistic or “biolistic” or gene gun technique consists of placing a nutrient agar plate carrying sections of *C. reinhardtii* cells (a lawn of microalgae cells) into a vacuum chamber and bombarding the plate with DNA-coated gold or tungsten microparticles. The produced energy by the particle bombardments is enough to penetrate the cell wall of *C. reinhardtii*, then the plasma membrane, reaches the two membranes surrounding the chloroplast, and finally delivers multiple copies of the transforming DNA into the organelle. This method can also be done with a cell-wall-deficient strain and with a quantity of DNA ranging from 1–2 μg [73].

### 5.4. Agrobacterium tumefaciens

The transformation of *C. reinhardtii* by *Agrobacterium* bacteria is a standard technique used initially for plants and then microalgae. In this method, *Agrobacterium tumefaciens* carry in its adequate plasmid (*pAgro* or *pCAMBIA*) the gene of interest to get integrated into *C. reinhardtii* cells. Once infected by the *Agrobacterium*, the microalgae cells integrate the gene of interest in their genome. Cells of *Agrobacterium* were prepared in advance in overnight culture and then incubated together with 10^7^ *C. reinhardtii* cells when OD_750nm_ is between 0.5 and 0.8 absorbance units. This method is not used yet for CRISPR-DNA transformation into *C. reinhardtii* [80]. Table 3 shows a comparative between the cited method of transformation for *C. reinhardtii* and their respective characteristics.

## 6. Applications of CRISPR Systems in *C. reinhardtii*

Since 2014, the exploration of CRISPR-Cas technology in *C. reinhardtii* has started. Jiang et al. [18] made the first integration’s attempt of CRISPR in the microalgae *C. reinhardtii* happening. However, this trial yields one positive rapamycin-resistant colony over a hundred of transformants and after 16 independent transformation experiments involving >10^9^ cells. The authors in this study explained that the failure to recover transformants with intact or expressed *Cas9* genes following transformation with the *Cas9* gene alone (or even with a gene encoding a Cas9 lacking nuclease activity) provided strong suggestive evidence for Cas9 toxicity when Cas9 is produced constitutively in *C. reinhardtii*. 

Two years later, two scientific articles from Korea revolutionized CRISPR science in *C. reinhardtii.* Shin et al. in 2016 [22] tried, for the first time, to use the Cas9 ribonucleoproteins (RNPs) delivery system, as cited previously. The Cas9 protein and sgRNAs helps to avoid cytotoxicity and off-targeting associated with vector-driven expression of Cas9. They obtained CRISPR-Cas9-induced mutations at three loci including *MAA7*, *CpSRP43*, and *ChlM*, and targeted mutagenic efficiency was improved up to 100-fold compared to the first report of transgenic Cas9-induced mutagenesis [18]. Consistent to that, the researchers also found that unrelated vectors used for the selection purpose were predominantly integrated at the Cas9 cut site, indicative of NHEJ-mediated knock-in events in the microalgae *C. reinhardtii*.

In the same year, Baek and collaborators [15] discussed the utilization of Cas9-sgRNA RNP in *C. reinhardtii*. This group targeted genes of the light-harvesting complex. This strategy allowed an easy visual selection based on the color of the algae. Another selection strategy of the efficacy of the CRISPR system has been then elucidated. There was no off-target in the genome of the microalgae, and the ribonucleic complex CRISPR-RNP demonstrated its success in modifying the genome of our algae of interest.

A year later, the German team Greiner et al. [14] described a procedure that creates antibiotic-resistant clones of *C. reinhardtii* after successful genetic modification, making the selection step much more rapid. Besides, they made several improvements to the current transformation protocols for both zinc-finger nucleases and CRISPR-Cas9 especially for the recovery process and light sensitivity. Their optimized methodologies allowed the disruption of photoreceptor genes and led to a better understanding of phototropism in *C. reinhardtii*. Greiner’s study yielded the first white colony of *C. reinhardtii* obtained by CRISPR modification and repaired by ZFN technology.

Indeed, genes of interest were disrupted in 5 to 15% of preselected clones (~1 out of 4000 initial cells), which was decent enough for these early studies.

In 2018, the first product-oriented application of the CRISPR system in *C. reinhardtii* was presented. In this study, the authors aimed to produce pigments and carotenoids via CRISPR-Cas9 genetic modification in the algae cells [23]. The targets were the two compounds: lutein and zeaxanthin. A knock-out mutant of the *zeaxanthin epoxidase* gene induced by preassembled DNA-free CRISPR-Cas9 RNPs in *C. reinhardtii* strain had a significantly higher zeaxanthin content (56-fold) and productivity (47-fold) than the wild type without the reduction in lutein level. Furthermore, they were able to produce eggs fortified with lutein (2-fold) and zeaxanthin (2.2-fold) by feeding hens a diet containing the mutant. Their results clearly demonstrate the possibility of cost-effective commercial use of microalgal mutants induced by DNA-free CRISPR-Cas9 RNPs in algal biotechnology to produce high-value products.

In 2019, Guzmán-Zapata and collaborators [20] demonstrated that transient expression of the *Streptococcus pyogenes* Cas9 gene and sgRNAs, targeted to the single-copy nuclear *apt9* gene, encoding an adenine phosphoribosyl transferase (*apt*), results in efficient disruption at the expected locus and then cells were able to grow in the presence of the toxin 2-FA. This transformation has been done via both glass beads agitation and particle bombardment. They obtained disruption efficiencies of 3 and 30% on preselected 2-FA resistant colonies, respectively. Their results showed that transient expression of Cas9 and sgRNA can be used for inexpensive and high-efficiency editing of the nuclear genome. Targeting of the *apt* gene could potentially be used as a pre-selection marker for multiplexed editing or disruption of genes of interest in the future. All these CRISPR applications are summarized in Table 4. 

## 7. Conclusions

Editing systems employing CRISPR-Cas is an emerging technology for green microalgae. Very recently, complete understanding and optimization of CRISPR-Cas made this biotechnological tool an interesting system to manipulate the genome of some freshwater and marine microalgae. Among two different classes and six distinct types of CRISPR systems, the Cas9-driven type II system has been the most used in CRISPR studies in microalgae, especially in the green microalga *C. reinhardtii*. Several discussed studies aimed to modulate the expression of desired genes in *C. reinhardtii*. Indeed, CRISPR technology that has been demonstrated in those previous researches may lead to the manifestation of particular gene functions and developing industrial traits, such as the production of yolks rich in carotenoids of high-added value.

Nevertheless, there are many components to be defined, enhanced, and optimized in *C. reinhardtii* to improve its efficiency. Challenges regarding the stability of the genetic edition and even slightly related specificity of targeted genome engineering should also be tackled shortly.

Overcoming those obstacles in *C. reinhardtii* CRISPR transformation will make this model microalgae a very efficient platform to produce therapeutic molecules, biofuels, recombinant proteins, or even vaccines. This will open the door to new areas of biotechnological applications based on the new microalgae toolbox CRISPR-Cas.

## Figures and Tables

**Table 1:** Cas9 and Cas12 Variants with Their Origin and Specifications.

Cas Protein	Bacterial Species	PAM Sequences	Used in *C. reinhardtii*	References
spCas9 native	*Streptococcus pyrogenes*	NGG	Yes	[35]
StCas9	*Streptococcus thermophilus*	NNAGAAW	Yes	[36]
saCas9	*Staphylococcus aureus*	NNGRRT or NNGRR(N)	Yes	[37]
nmCas9	*Neisseria meningitidis*	NNNNGATT	Not yet	[36]
Cpf1 (Cas12)	*Prevoltella* and *francisella 1*	NTT	Yes	[38]
dCas9 (dead)	Engineered *S. pyrogenes*	NGG	Not yet	[39]
nCas9 (nikase)	Engineered *S. pyrogenes*	NGG	Not yet	[40]

**Table 2:** Representative Support Tools for sgRNA Design.

Tool Name	Species	Website	References
CRISPRko	Human and mouse.	https://portals.broadinstitute.org/gpp/public/analysis-tools/sgrna-design	[41]
Cas-OFFinder	Vertebrates. Insects, plants, fungi, prokaryotes, *C. reinhardtii*	http://www.rgenome.net/cas-offinder/	[42]
CRISPOR	Vertebrates, insects, plants, fungi, prokaryotes.	http://crispor.tefor.net/	[43]
E-CRISP	Vertebrates, invertebrates. Plants, fungi, protists, and *C. reinhardtii.*	http://www.e-crisp.org/E-CRISP/aboutpage.html	[44]
Off–spotter	Human, mouse, fungi	https://cm.jefferson.edu/Off-Spotter/	[45]
CRISPR-GE	Vertebrates, invertebrates, plants	http://skl.scau.edu.cn/targetdesign/	[46]
Benchling	Various: vertebrates. Insects, plants, fungi, prokaryotes, and *C. reinhardtii*	https://benchling.com/	-
Deskgen	Various: vertebrates. Insects, plants, fungi, prokaryotes	https://www.deskgen.com/	[47]
CHOPCHOP	Various: vertebrates. Insects, plants, fungi, prokaryotes and *Chlamydomonas*	https://chopchop.rc.fas.harvard.edu/	[48]
CRISPR MultiTargeter	Various: vertebrates. Insects, plants, fungi, prokaryotes	http://www.multicrispr.net/	[49]
sgRNA Scorer	Various: vertebrates. Insects, plants, fungi, prokaryotes.	https://crispr.med.harvard.edu/sgRNAScorerV1/	[50]
CRISPRscan	Various: vertebrates. Insects, plants, fungi, prokaryotes.	http://www.crisprscan.org/	[51]

**Table 3:** Summary of the Most Used Methods of Transformation of *C. reinhardtii* Cells and Their Characteristics.

Methods of Transformation	DNA Type	DNA Quantity	Cells Quantity	Cells Type	Plasmid Size	Efficiency of Transformation	References
Electroporation	Not CRISPR	100 ng	1.0–2.0 × 10^7^ cells ml^−1^	WT	>10 Kb	0.4–3 × 10^3^ per μg	[76]
	CRISPR	2–4 µg	100× 10^6^	Wall-less strain	Not mentioned	31–367	[81]
Glass beads	Not CRISPR	1–3 µg	5 × 10^5^ or OD_750_ nm: 0.5–0.8	Preferably cell wall-less cells but also WT	Up to 10 Kb	Low (10^3^–10^4^)	[82]
	CRISPR	3 µg	2 × 10^8^ cells			0.5 × 10^8^	[20]
Biolistic	Not CRISPR	1–3 µg/µL	10^6^ to 10^7^ cells/mL	Cell wall deficient strain	Up to 10 Kb	Low 300–600/3 µg of DNA	[73]
	CRISPR	1 μg/µL	1 × 10^8^ cells	Wild type		0.5 × 10^8^	[20]
*A. tumefaciens*	Not CRISPR	Bacterial culture 40–200 µL	10^8^ cells/mL	Cell wall deficient strains	>10 Kb, specific plasmid	10^8^	[80]

**Table 4:** Summary of the Main Articles Related to CRISPR-Cas Transformation in *C. reinhardtii* and Valuable Information Regarding the Technical Aspects.

Author and Year	System	Targeted Gene	Codon Optimization	Cas	Proof of Concept	*C. reinhardtii* Strain	Efficiency of Mutations	Transformation Method	Off Target	Antibiotics
Jiang et al., 2014 [18]	knock-out to disrupt *FKB12* gene	*FKB12*: *C. reinhardtii* cell lacking *FKB12* are fully resistant to rapamycin antibiotics	yes	Cas9 and dCas9 as control of toxicity	Cells becoming resistant to rapamycin	CC-503	Only one colony and failure to recover more transformants	electroporation	not studied	rapamycin
Baek et al., 2016 [15]	knock-out using DNA-free CRISPR-Cas9 method, disrupting *CpFTSY* and ZEP and generating a strain producing zeaxanthin	The knockout of the *CpFTSY* gene confers smaller, or truncated, chlorophyll (Chl) antenna size of the photosystems. The knockout of zeaxanthin epoxidase (*ZEP*) leads to constitutive accumulation of zeaxanthin	yes	Cas9	*CpFTSY*: pale green color *ZEP*: zeaxanthin accumulation *CpFTSY/ZEP*: zeaxanthin and golden color	CC-4349 cw15 mt-	*CpFTSY*:0.56% *ZEP*: 0.46%	electroporation	*CpFTSY*: far up to 4 nucleotides from the on-target *ZEP*: no off-target	no antibiotics used
Sung shin et al., 2016 [22]	Complex RNP for knock-out, plasmids *CpSRP43* and *pCr202*	*MAA7* encodes for the beta subunit of tryptophan synthase	yes	Cas9	5-fluoro-indole	CC-124	40%	electroporation	no off-target events	no antibiotics used
		*CpSRP43*: antenna assembly genes encode for chloroplast			Light colony color and low chlorophyll dosage		1.4%			
		*ChLM*: when disrupted affects chlorophyll biosynthesis and reduces the accumulation of major thylakoid-associated proteins					1.7%			
Greiner et al., 2017 [14]	RNP and plasmid knock-out of *PSY1*	*PSY1*: Phytoene synthase 1 catalyze carotenoids synthesis and photosynthesis	yes	Cas9	white colony	CC-125	not mentioned	electroporation	no off-target events	paromomycin antibiotics
Baek et al., 2017 [23]	Knock-out of zeaxanthin epoxidase with RNP CRISPR	*ZEP: zeaxanthin epoxidase*, when disrupted the microalgae accumulates zeaxanthin	-	Cas9	zeaxanthin dosage	CC-4349, CC-124, CC-620, CC-4051, CC-4533	not mentioned	glass beads	no off-target (Cas-OFFinder, reported in [15])	no antibiotics used
Ferenczi et al., 2017 [21]	RNP CRISPR for the gene *Fkb12* with a repair plasmid ssODN	*Fkb12*: mediates the interaction between the antibiotic rapamycin and the cell cycle regulator target of rapamycin, which leads to cell death.	yes	*Lb*CPpf1	high tolerance to rapamycin	CC-1883, CC-2931	29%	electroporation	no predicted off-target sites with Cas-ORFinder	Rapamycin
Findinier et al., 2019 [83]	RNP CRISPR for the gene *CrFzI*	*CrFzi*: phenotypic analyses revealed a specific requirement of *CrFzl* for survival upon light stress. Consistent with this, strong irradiance leads to increased photoinhibition of photosynthesis in mutant cells	not mentioned	Cas9	when *Fzi* is disrupted, it promotes thylakoid fusion and resistance to light stress	cc-4533, cw15.J3	not mentioned	electroporation	not mentioned	Hygromycin resistant gene
Guzmán-Zapata et al., 2019 [20]	plasmid CRISPR	*Apt*: sensitivity to a toxin 2-fluoroadenine (2-FA)	Yes	Cas9	growth on the 2-FA toxin	CC-125, CC-3403, SAG73.72, CC-3403-*uvr*8-2	not mentioned	glass beads/particle bombardment	not mentioned	no antibiotic used
Kim et al., 2020 [84]	knock- out RNP CRISPR Cas9 and knock-in antibiotics resistance gene and *YFP*	*CrFTSY*: confers smaller, or truncated, chlorophyll (Chl) antenna size of the photosystems ([15])	not mentioned	Cas9	pale green color and measurement of luciferase activity	CC-4349, CC-124, and CC-503	up to 30%	electroporation	not mentioned	hygromycin, paromomycin
